# Epidemiology and Visual Outcome of Open Globe Injury Cases in Hospital Pulau Pinang

**DOI:** 10.7759/cureus.19648

**Published:** 2021-11-16

**Authors:** Wang Shir Yen, Foo Siu Wan, Jemaima Che Hamzah, Karen Khoo Kah Luen

**Affiliations:** 1 Department of Ophthalmology, Faculty of Medicine, Universiti Kebangsaan Malaysia, Kuala Lumpur, MYS; 2 Department of Ophthalmology, Hospital Pulau Pinang, Ministry of Health Malaysia, George Town, MYS

**Keywords:** penang, ocular trauma score (ots), visual outcome, open globe injury, epidemiology

## Abstract

Objective

To describe the epidemiology and to evaluate the visual outcome of open globe injury (OGI) cases in Hospital Pulau Pinang.

Method

A three-year retrospective study on OGI cases presenting to Hospital Pulau Pinang from January 2018 until December 2020.

Result

A total of 39 OGI cases (n=39) were included in this study. The average age of the patients was 34.9 ± 21.7 (mean ± standard deviation, SD). There were 33 males (84.6%) and six females (15.4%). In this study, 27 cases (69.2%) were Malaysians, while the remaining 12 cases (30.8%) were foreigners. OGI cases were mostly caused by occupational injuries (n=17, 43.6%) and domestic-related accidents (n=17, 43.6%). The mean initial VA (visual acuity) logMAR was 1.69 ± 0.98 (mean ± SD). Overall, the final VA improved to the mean VA logMAR of 1.04 ± 1.08 (mean ± SD). There was a significant positive correlation between initial VA and final VA logMAR (Spearman’s rho = 0.6532, p <0.001). A negative linear correlation was found between calculated raw points of Ocular Trauma Score (OTS) and final VA logMAR (Spearman’s rho = -0.7067, p <0.001).

Conclusion

Young adult males, foreign nationality, occupational injuries, and domestic-related accidents are risk factors of OGI. By uncovering the risk, we can take remedial actions to ensure better public health and clinical strategies to prevent and manage ocular trauma in the future. This study also highlights that initial VA and OTS are effective in predicting visual outcomes of OGI.

## Introduction

Ocular trauma is one of the preventable causes of visual impairment and blindness globally. WHO proposed that there are 55 million eye injuries restricting activities for over a day annually, among which 200000 cases are perforating eye injuries, thus accounting for a global incidence rate of 3.5 per 100000 population. This phenomenon leads to blindness in 1.6 million eyes and low vision in 2.3 million eyes bilaterally. It also accounts for almost 19 million unilateral blindness or low vision [1]. Apart from its ocular morbidity, ocular trauma also has inevitable impacts on the quality of life, mental health, and financial burden on patients, employers, and the country. Ocular trauma has a wide range of clinical presentations and can be divided into open and closed globe injuries. The Birmingham Eye Trauma Terminology (BETT) System was created by Kuhn et al. to standardize the ocular trauma terminology for each type of injury. By definition, open globe injury (OGI) is a full-thickness laceration wound of the eye wall [2]. It can be further classified into subtypes of rupture, penetration, perforation, and intraocular foreign body (IOFB). OGI is an ocular emergency that frequently requires a more extended hospital stay and carries a poorer visual prognosis as compared to closed globe injury [3-6]. In recent years, many studies related to ocular trauma have been conducted internationally and locally because of the increasing public health awareness worldwide [7-10]. This study was conducted to describe the epidemiology and nature of OGI cases in Hospital Pulau Pinang. It also aims to evaluate the correlation between the visual outcome of OGI and initial visual acuity as well as the Ocular Trauma Score (OTS). 

## Materials and methods

This is a three-year retrospective study on medical records of OGI cases presented to Hospital Pulau Pinang from January 2018 until December 2020. Classification of OGI was based on the Birmingham Eye Trauma Terminology (BETT). Patients were identified from operating theatre records to retrieve their case notes. Information such as demographic profile, characteristics of ocular injuries, management, and the visual outcome was recorded. Both initial visual acuity (VA) and final visual acuity were categorised into no perception of light (NPL), perception of light or hand movement (PL/HM), 1/200-19/200, 20/200-20/50, and ≥20/40. Both VA were then converted to log MAR for the purpose of analysis [11]. Retrospectively, we calculated the raw points of Ocular Trauma Score (OTS) at presentation based on certain numerical values (initial VA, rupture, endophthalmitis, perforating injury, retinal detachment, and afferent pupillary defect) proposed by Kuhn et al. to predict the visual outcome [12]. Correlations between initial VA, raw points of OTS, and final VA were analysed using Spearman’s test. Data analysis was done using SPSS Statistics v. 26 (IBM Corp, Armonk, NY).

## Results

Demographics and presenting ocular characteristics of open globe injury

A total of 39 OGI cases (n=39) were included in this study (Table 1). All cases had unilateral involvement where both eyes had almost equal distribution, with 16 cases involving the right eye (41.0%) and 23 cases involving the left eye (59.0%). Overall, the average age of the patients was 34.9 ± 21.7 (mean ± standard deviation, SD). There were 33 males (84.6%) and six females (15.4%), with a male-to-female ratio of 5.5:1 (Table 2). In this study, 27 cases (69.2%) were Malaysians, while the remaining 12 cases (30.8%) were foreigners. Among Malaysians, Malay was the predominant race (n=16, 41.0%), followed by Chinese (n=8, 20.5%) and lastly Indian (n=3, 7.7%). OGI cases were mostly caused by occupational injuries (n=17, 43.6%) and domestic-related accidents (n=17, 43.6%), followed by motor vehicle accidents (n=4, 10.3%) and one assault case (n=1, 2.6%). Of all occupational injuries, 14 cases were related to high-velocity projectile activities such as hammering (n=8), grinding or cutting metal (n=4), welding (n=1) and blast injury (n=1), while three other cases were not specified. Regarding offending objects implicated at the workplace, the metal piece was the most common cause (n=7), followed by nails (n=4), and others such as glass, rock, tile, wire, screwdriver, blade from grinding machine. Among occupational injuries, we noticed a male preponderance (n=17, 100%). The racial distribution in occupational injuries had the highest incidence among foreigners (n=10, 58.8%), then Malay (n=4, 23.5%), Indian (n=2, 11/8%), and lastly Chinese (n=1, 5.9%). Among all the cases, 28 cases were penetrating injury (71.8%), eight were rupture (20.5%), and three were IOFB (7.7%). Other concomitant ocular problems at the time of presentation such as rupture (n=8), positive afferent pupillary defect (APD) (n=5), retinal detachment (n=4) and endophthalmitis (n=1) were observed. Majority of the patients (n=28, 71.8%) presented to the hospital within 24 hours from the onset of the injury. All the patients were hospitalised, with an average of 4.5±2.87 days of hospital stay (mean ± SD).

**Table 1:** Demographics and presenting ocular characteristics of open globe injury, n=39 SD = standard deviation; IOFB = intraocular foreign body; RD = retinal detachment; APD = afferent pupillary defect; OTS = Ocular Trauma Score; VA = visual acuity

Demographics and Presenting Ocular Characteristics of Open Globe Injury	n (%)
Age, mean (SD)	34.9 (21.75)
Gender	
Male	33 (84.6)
Female	6 (15.4)
Race	
Malay	16(41.0)
Chinese	8 (20.5)
Indian	3 (7.7)
Foreigner	12 (30.8)
Trauma eye	
Right	16 (41.0)
Left	23 (59.0)
Causes of injury	
Occupational injuries	17 (43.6)
Domestic-related accidents	17 (43.6)
Motor vehicle accidents	4 (10.3)
Assault cases	1 (2.6)
Time to injury presentation	
<24 hours	28 (71.8)
24 – 48 hours	2 (5.1)
48 – 96 hours	6 (15.4)
>96 hours	3 (7.7)
Types of open globe injury	
Rupture	8 (20.5)
Penetrating	28 (71.8)
IOFB	3 (7.7)
Concomitant ocular problems at presentation	
Endophthalmitis	1 (2.6)
RD	4 (10.3)
Rupture	8 (20.5)
APD	5 (12.8)
Days of hospitalization, mean (SD)	4.5 (2.87)
Mean OTS score (SD)	71.1 (20.0)
OTS	
1	3 (7.7)
2	11 (28.2)
3	13 (33.3)
4	7 (17.9)
5	5 12.8)
Mean Initial VA log MAR (SD)	1.69 (0.98)
Mean Final VA log MAR (SD)	1.04 (1.08)

**Table 2:** Relationship between causes of injury and gender ^1^ Fisher’s exact test

Causes of injury	Gender		p-value^1^
	Male, n (%)	Female, n (%)	
Occupational injuries	17 (51.5)	0	0.028
Domestic-related accidents	11 (33.3)	6 (100)	
Motor vehicle accidents	4 (12.12)	0	
Assault cases	1 (3.0)	0	

Management of Open Globe Injury

Primary ocular wall closure was conducted in all the cases (n=38) except one case, which was managed conservatively (Table 3). In more than half of the cases, primary ocular wall closure was done to cornea and limbus (n=23, 58.9%), while the rest involved the closure of sclera (n=9, 23.1%) and corneoscleral (n=6, 15.4%). Besides primary repair, surgical procedures such as lens removal, posterior vitrectomy, removal of IOFB, intravitreal antibiotics, and anterior chamber washout were performed on a case-by-case basis. Half of the lens removal cases were done in the primary setting (six among 12 cases), while all posterior vitrectomy cases were performed in the secondary setting (n=2).

**Table 3:** Management of Open Globe Injury, n=39 IOFB = intraocular foreign body; AC = anterior chamber

Type of management	n (%)
Primary ocular wall closure	
None	1 (2.6)
Cornea and Limbus	23 (58.9)
Sclera	9 (23.1)
Corneo-sclera	6 (15.4)
Lensectomy/ Phacoemulsification	12 (30.8)
Primary Lensectomy/ Phacoemulsification	6 (15.4)
Secondary Lensectomy/ Phacoemulsification	6 (15.4)
Posterior Vitrectomy	2 (5.1)
Removal of IOFB	3 (7.7)
Intravitreal Antibiotic	17 (43.6)
AC Washout	6 (15.4)

Initial visual acuity, OTS, final visual acuity and their associations

**Figure 1:**
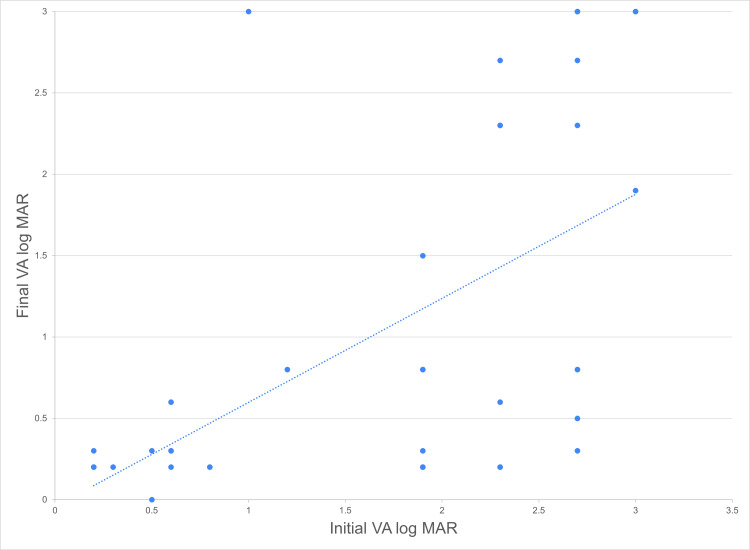
Scatter plot showing the distribution of cases (n=39) according to the final VAlog MAR and initial VA logMAR (Spearman’s rho = 0.6532, p <0.001)

**Figure 2:**
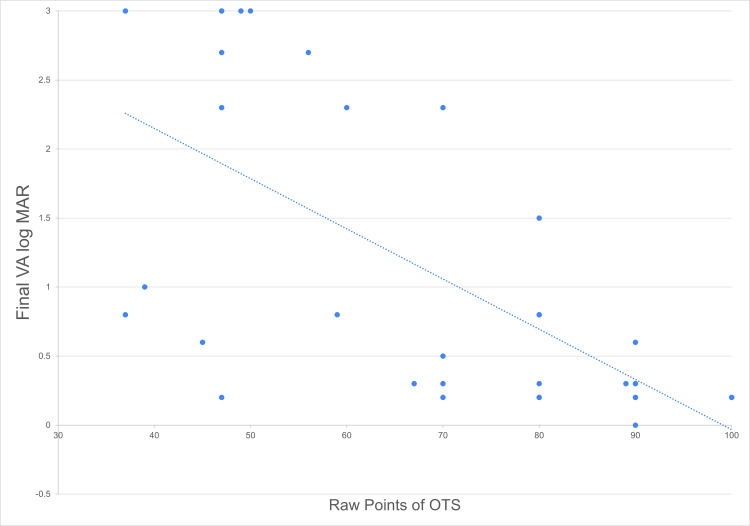
Scatter plot showing the distribution of cases (n=39) according to the final VA log MAR and raw points of OTS (Spearman’s rho = -0.7067, p <0.001).

At the time of presentation, majority of initial VA were poor with PL/HM (n=14, 35.9%), followed by 1/200-19/200 (n=9, 23.1%), 20/200-20/50 (n=8, 20.5%), ≥20/40 (n=5, 12.8%), and lastly NPL (n=3, 7.8%). The mean initial VA log MAR was 1.69 ± 0.98 (mean ± SD). The best-corrected VA (BCVA) at last follow-up was considered as the final VA. Overall, there was an improvement in the final VA with the mean VA log MAR of 1.04 ± 1.08 (mean ± SD). Almost half of the cases achieved final VA of ≥20/40 (n=19, 48.5%), followed by 20/200-20/50 (n=8, 20.5%), PL/HM (n-5, 12.5%), NPL (N-5, 12.5%) and lastly 1/200-19/200 (n=2, 5.1%) (Table 4). There was a significant positive correlation between initial VA and final VA logMAR (Spearman’s rho = 0.6532, p <0.001), as illustrated in Figure 1. Besides, a negative linear correlation was found between calculated raw points of OTS and final VA logMAR (Spearman’s rho = -0.7067, p <0.001), as illustrated in Figure 2. Based on the raw points of OTS, all cases were further categorised into categories one to five. The percentage of the final VA by OTS category is shown in Table 5. In conclusion, the final VA was found to be significantly associated with the initial VA and OTS.

**Table 4:** Initial and final VA by category NPL= No perception of light; PL = perception of light; HM = hand movement

	Initial VA, n (%)	Final VA, n (%)
NPL	3 (7.8)	5 (12.5)
PL/HM	14 (35.9)	5 (12.5)
1/200-19/200	9 (23.1)	2 (5.1)
20/200-20/50	8 (20.5)	8 (20.5)
≥20/40	5 (12.8)	19 (48.7)

**Table 5:** Percentage of the final VA by OTS category NPL= No perception of light; PL = perception of light; HM = hand movement

Raw points of OTS	OTS	NLP	PL/HM	1/200-19/200	20-200-20/50	≥20/40	Total
		n (%)	n (%)	n (%)	n (%)	n (%)	n (%)
0-44	1	1 (33.3)	0 (0.0)	1 (33.3)	1 (33.3)	0 (0.0)	3 (100.0)
45-65	2	4 (36.4)	4 (36.4)	0 (0.0)	2 (18.2)	1 (9.1)	11 (100.0)
66-80	3	0 (0.0)	1 (7.7)	1 (7.7)	4 (30.8)	7 (53.8)	13 (100.0)
81-91	4	0 (0.0)	0 (0.0)	0 (0.0)	0 (0.0)	7 (100.0)	7 (100.0)
92-100	5	0 (0.0)	0(0.0)	0 (0.0)	1(20.0)	4(80.0)	5 (100.0)

 

## Discussion

Hospital Pulau Pinang is a public hospital situated in the city of George Town. It is a tertiary referral hospital catering to a population of around 800,000 in Penang Island [13]. Besides Hospital Pulau Pinang, there are several private eye specialist centres in the region which may explain the small sample size in our study. Therefore, this study may not reflect the entire population in this region. However, the demographic profiles in our data agreed with findings in other local and international studies.

Young adults with a mean age of 34.9 are the population at risk, corroborated with other OGI studies conducted in Malaysia and other countries with the mean range of 30 to 39.1 [7-8, 14-15]. Consistently, many studies showed a higher incidence of ocular trauma among males than females [14-18]. This situation could be explained by gender-based aggressive behaviour and a higher proportion of male involvement in accident-prone work. All the female cases in this study were related to domestic-related accidents, similarly reported by Omar et al. and Koo et al. [10, 17]. This scenario can be partially attributed to a significant proportion of housewives or higher unemployment rates among the female population in Malaysia, as reported in the Fifth Malaysian Population and Family Survey [19].

Our study showed a high incidence of OGI caused by occupational injuries and domestic-related accidents. Ocular injury at the workplace is an important cause of visual impairment observed in many previous studies conducted locally and globally [7-8, 10, 14, 16, 20]. In Malaysia, foreigners accounted for a significant number of OGI cases, with the majority related to occupational injuries [10, 21]. This phenomenon shows parallels to our study, where most of them migrate from neighbouring countries such as Indonesia, Nepal, and Bangladesh. They spoke different native languages and usually had low education levels. Language barrier and difficulties in understanding the health and safety measures at their workplace may explain the high incidence of OGI [7]. Most foreigners were involved in agricultural, manufacturing, and construction sectors, which are relatively high-risk jobs [22-23]. Despite the high incidence of ocular trauma at the workplace, Mallika et al. reported that none of the workers were wearing protective eye devices at the time of injury [8]. A similar finding was observed by Soong et al [7]. Hence, employers should educate workers on safety measures to increase compliance with protective eye devices. Increments in financial penalties to employers who violate the law may also reduce the incidence, as shown in a study conducted in the United States [24]. Thus, strict rules on health and safety at the workplace should be enforced as a preventive measure. Besides, few studies also showed a high prevalence of ocular trauma which occurred at home [8-9, 25]. OGI at home can be related to activities like playing, “do-it-yourself” activities, and falls [9]. Home improvement and “do-it-yourself” activities involving dangerous tools were commonly carried out at home with no professional skills or training [7]. Adding to that, the lack of protective eyewear may increase the occurrence of ocular trauma. Under the group of domestic-related accidents, we noticed 10 among 17 cases were children below 12 years of age [4]. This phenomenon highlights the importance of close supervision of children at home by parents or caretakers and identifying potentially dangerous objects at home.

Public health awareness among cases in this study was high as 71.8% of cases sought medical treatment promptly within one day from the onset of the injury. This may be attributed to other logistic factors such as the small geographical area of the region and the availability of transport systems. Regarding management of OGI, most of our cases underwent primary ocular wall closure on cornea and limbus, corresponding to Zone I as defined in BETT. Most of the previous studies reported that OGI confined to the cornea carries a better visual prognosis compared to those involving the sclera [9-10, 20, 26]. This can be helpful to create training modules on surgical management of OGI for junior medical officers and ophthalmologists.

In this study, initial VA is significantly correlated to final VA as observed in other OGI studies [9-10, 14-15, 20, 27]. We also demonstrated that a higher OTS category correlates with better final VA, consistent with the OTS study [12]. All patients in OTS category four and 80% of category five successfully achieved final VA ≥20/40, contrary to only 53.8%, 9.1%, and none in category three, two, and one, respectively. Overall, the initial VA and the objective scoring system are effective in forecasting the visual outcome. This prediction can be helpful for clinicians to decide on the management as well as to provide realistic expectations to patients during counselling.

Several limitations were found in this study, such as insufficient information mainly because of the study’s retrospective nature and the small sample size.

## Conclusions

Open globe injury is an important ocular emergency. To summarize, young adult males, foreign nationality, occupational injuries, and domestic-related accidents are important risk factors. By uncovering the risks, we can take remedial actions to ensure the better public health and clinical strategies to prevent and manage ocular trauma in the future. This study also highlights that initial VA and OTS are effective in predicting the visual outcome of OGI.

## References

[1] Négrel AD, Thylefors B (1998). The global impact of eye injuries. Ophthalmic Epidemiol.

[2] Kuhn F, Morris R, Witherspoon CD (2002). Birmingham Eye Trauma Terminology (BETT): terminology and classification of mechanical eye injuries. Ophthalmol Clin North Am.

[3] Kinderan YV, Shrestha E, Maharjan IM, Karmacharya S (2012). Pattern of ocular trauma in the western region of Nepal. Nepal J Ophthalmol.

[4] Al-Mahdi HS, Bener A, Hashim SP (2011). Clinical pattern of pediatric ocular trauma in fast developing country. Int Emerg Nurs.

[5] Soylu M, Sizmaz S, Cayli S (2010). Eye injury (ocular trauma) in southern Turkey: epidemiology, ocular survival, and visual outcome. Int Ophthalmol.

[6] Onakpoya OH, Adeoye A, Adeoti CO, Ajite K (2010). Epidemiology of ocular trauma among the elderly in a developing country. Ophthalmic Epidemiol.

[7] Soong TK, Koh A, Subrayan V, Loo AV (2011). Ocular trauma injuries: a 1-year surveillance study in the University of Malaya Medical Centre, Malaysia. 2008. Graefes Arch Clin Exp Ophthalmol.

[8] Mallika P, Tan A, Asok T, Faisal H, Aziz S, Intan G (2008). Pattern of ocular trauma in kuching, malaysia. Malays Fam Physician.

[9] Madhusudhan AP, Evelyn-Tai LM, Zamri N, Adil H, Wan-Hazabbah WH (2014). Open globe injury in Hospital Universiti Sains Malaysia - A 10-year review. Int J Ophthalmol.

[10] Omar N, Aim MA, Ismail R, Saleh RM (2017). Open globe injury in Hospital Serdang - an 8-year retrospective review. Malaysian J Med Health Sci.

[11] Patel H, Congdon N, Strauss G, Lansingh C (2017). A need for standardization in visual acuity measurement. Arq Bras Oftalmol.

[12] Kuhn F, Maisiak R, Mann L, Mester V, Morris R, Witherspoon CD (2002). The Ocular Trauma Score (OTS). Ophthalmol Clin North Am.

[13] (2021). Penang’s population and demographics. https://public.tableau.com/views/Penangspopulationanddemographics/Overalltrend?:embed=y&:showVizHome=no&:host_url=https%3A%2F%2Fpublic.tableau.com%2F&:embed_code_version=3&:tabs=yes&:toolbar=yes&:animate_transition=yes&:display_static_image=no&:display_spinner=no&:display_overlay=yes&:display_count=yes&:loadOrderID=0.

[14] Supreeyathitikul P, Chokesuwattanaskul S, Choovuthayakorn J, Patikulsila D, Watanachai N, Kunavisarut P, Chaikitmongkol V (2020). Epidemiology and outcomes following open globe injury in agricultural region, an 11-year experience. Ophthalmic Epidemiol.

[15] Ozturk T, Cetin Dora G, Ayhan Z, Kaya M, Arikan G, Yaman A (2019). Etiology and visual prognosis in open globe injuries: results of a tertiary referral center in Turkey. Sci Rep.

[16] Zhang X, Liu Y, Ji X, Zou Y (2017). A retrospective study on clinical features and visual outcome of patients hospitalized for ocular trauma in Cangzhou, China. J Ophthalmol.

[17] Koo L, Kapadia MK, Singh RP, Sheridan R, Hatton MP (2005). Gender differences in etiology and outcome of open globe injuries. J Trauma.

[18] Hooi SH, Hooi ST (2003). Open-globe injuries: the experience at Hospital Sultanah Aminah, Johor Bahru. Med J Malaysia.

[19] National Population and Family Development Board (2016). [19] National Population and Family Development Board (NPFDB), Ministry of Women, Family and Community Development, Malaysia, and Lembaga Penduduk dan Pembangunan Keluarga Negara (LPPKN), Report on key findings Fifth Malaysian Population and Family Survey (MPFS-5). Report on Key Findings - Fifth Malaysian Population and Family Survey (MPFS-5).

[20] Thevi T, Mimiwati Z, Reddy SC (2012). Visual outcome in open globe injuries. Nepal J Ophthalmol.

[21] Min NN, Vasudevan SK, Jasman AA, Aisyahbinti A, Myint KT (2016). Work-related ocular injuries in Johor Bahru, Malaysia. Guoji Yanke Zazhi.

[22] (2021). Malaysia - Estimating the number of foreign workers (a report from the Labor Market Data for Monetary Policy task). World Bank. https://documents1.worldbank.org/curated/en/953091562223517841/pdf/Malaysia-Estimating-the-Number-of-Foreign-Workers-A-Report-from-the-Labor-Market-Data-for-Monetary-Policy-Task.pdf.

[23] Devadason ES, Meng CW (2014). Policies and laws regulating migrant workers in Malaysia: a critical appraisal. J Contemp Asia.

[24] McCall BP, Horwitz IB, Taylor OA (2009). Occupational eye injury and risk reduction: Kentucky workers' compensation claim analysis 1994-2003. Inj Prev.

[25] Desai P, Morris DS, Minassian DC, MacEwen CJ (2015). Trends in serious ocular trauma in Scotland. Eye (Lond).

[26] Makhrash MA, Gosadi IM (2016). Open globe eye injury characteristics and prognostic factors in Jazan, Saudi Arabia. Saudi Med J.

[27] Court JH, Lu LM, Wang N, McGhee CN (2019). Visual and ocular morbidity in severe open-globe injuries presenting to a regional eye centre in New Zealand. Clin Exp Ophthalmol.

